# Validation of measurement scores for evaluating vascular anomaly skin lesions

**DOI:** 10.1111/1346-8138.15839

**Published:** 2021-03-30

**Authors:** Shiho Yasue, Michio Ozeki, Saori Endo, Takuma Ishihara, Mana Nishiguchi‐Kurimoto, Masatoshi Jinnin, Miho Kawamura, Mariko Seishima, Hidenori Ohnishi

**Affiliations:** ^1^ Department of Pediatrics Graduate School of Medicine Gifu University Gifu Japan; ^2^ Innovative and Clinical Research Promotion Center Graduate School of Medicine Gifu University Gifu Japan; ^3^ Department of Dermatology Graduate School of Medicine Gifu University Gifu Japan; ^4^ Department of Dermatology Wakayama Medical University Wakayama Japan

**Keywords:** disease scoring system, drug treatment, lymphatic malformation, vascular tumor, venous malformation

## Abstract

Vascular anomalies comprise a heterogeneous group of disorders caused by abnormal proliferation or development of vascular and/or lymphatic vessels. Vascular anomalies present with various symptoms and complications, but no standardized methods evaluate their severity, and to measure treatment outcomes is difficult. To assess the responsiveness of measurement scores for evaluating vascular anomaly skin lesions, we conducted a validation study to compare these measurement scores with patients’ objective data. In this study, data were collected from treated and untreated patients. Skin lesions were photographed at baseline and after a follow‐up period of 3–6 months. The volume of skin lesions, the degree of red or purple coloration, and color tone were measured objectively. Two external dermatologists evaluated patients’ photographs and determined scores, which represented criteria for improvements in skin lesions (size and color) and 6‐point Physician Global Assessment scores. The correlation between these scores and patients’ objective data (lesion volume and color) was assessed to validate the scores. Twenty‐three cases of vascular anomaly (seven vascular tumors, five lymphatic malformations, three venous malformations, and eight lymphatic–venous malformations) were examined. Scores for improvements in vascular anomaly skin lesions (size and color) correlated with a change in lesion volume, the degree of red or purple coloration, color tone score, and 6‐point Physician Global Assessment score. Our findings suggest that these measurement scores are responsive to changes in vascular anomaly skin lesions after observation.

## 1 INTRODUCTION

Vascular anomalies (VA) include many heterogeneous disorders that frequently occur in childhood.^1^ A classification from the International Society for the Study of Vascular Anomalies divided VA into vascular tumors (VT) and vascular malformations.^2^ Infantile hemangiomas (IH) are common and are generally not complicated; however, other VT, including tufted angiomas (TA) and kaposiform hemangioendotheliomas (KHE), are very rare and have poor outcomes because they can cause severe coagulopathy (the Kasabach–Merritt phenomenon). Slow‐flow vascular malformations involve abnormal venous vessels (venous malformations [VM]), lymph vessels (lymphatic malformations [LM]), and a combination of these (combined vascular malformations). Management of these diseases requires multidisciplinary care, but no standardized treatments exist at present.^1^ Most patients manifest with disease in the superficial, cutaneous, or subcutaneous tissues of the face, whole body, and limb, which may cause major concerns with appearance. Treatments for these patients include surgical resection, laser therapy, and sclerotherapy; however, these treatment methods are not curative.

Recently, mammalian target of rapamycin (mTOR) inhibitors, such as sirolimus, have been used to treat VA with promising curative results.^3^, ^4^, ^5^ However, currently no validated methods evaluate treatment outcomes of novel drugs. Recent research used divergent methods to assess changes in lesions, such as radiological responses measured by magnetic resonance imaging, pain measured using the visual analog scale, and quality of life (QOL) scores.^3^, ^4^, ^5^ Although many assessment methods have been used in clinical practice, no standardized methods assess VA because these diseases are heterogeneous, have different clinical presentations, and affect distinct anatomical areas. With VA skin lesions, it is extremely difficult to unitarily analyze disparate symptoms. Therefore, a new scoring system to evaluate changes in VA lesions is needed to develop novel treatments.

In this study, we aimed to elucidate optimal measurements to evaluate the efficacy of novel treatments for cutaneous VA. Therefore, we validated measurement scores, which we modified by referring to past scores for other diseases, by comparing subjective assessments evaluating the size and color of skin lesions with objective assessments.

## 2 METHODS

### 2.1 Study design, ethical approval, patient enrollment, and data collection

This study adopted a retrospective design to validate measurement scores for evaluating VA skin lesions. The institutional review board of Gifu University Graduate School of Medicine approved this study, which was registered in November 2020 (trial registration no. UMIN000042388). Patients with VA (TA, KHE, VM, LM, and combined vascular malformations) who had skin lesions and were examined at Gifu University Hospital from 1 January 2015 to 31 December 2020, were enrolled. Patients who received drug therapy for VA were included, but patients who had previously undergone surgical resection or laser therapy and patients with infected lesions were excluded.

Attending physicians collected patients’ data (type of VA, age, sex, lesion location, and treatment) and evaluated the size (volume) and color of skin lesions. Patients’ photographs, which were taken by one photographer under comparable conditions (distance, location, and camera), were taken at baseline (Visit 1) and after a follow‐up period of 3–6 months (Visit 2). Volume was determined using the major axis, minor axis, and thickness. Color was quantified by measuring the degree of red or purple coloration (Pantone^®^ Color Sample (Pantone LLC, Carlstadt, NJ, USA)) (Tables S1 and S2). To objectively calculate the gray value of the affected skin lesion, three regions of interest (ROI) of lesions and normal skin in each photograph were selected, and the mean gray values of ROI were measured using image analysis software (Image J (US National Institutes of Health, Bethesda, MD, USA)). The skin lesion color value was determined as the ratio of cutaneous lesion color to normal skin color.

### 2.2 Assessment

To ensure assessment score reliability, two dermatologists who were independent from other investigators performed the assessment. These external evaluators evaluated patients’ photographs and determined scores, which were used as criteria for improvements in VA skin lesions (size and color) (Table 1), VA size, and VA color (Tables S3 and S4). These measurement scores were created based on previously reported scores for facial angiofibroma in patients with tuberous sclerosis complex (TSC).^6^ These criteria are categorized on a 6‐point scale: “marked” (score of 3), “improved” (score of 2), “slightly improved” (score of 1), “unchanged” (score of 0), “slightly exacerbated” (score of −1), and “exacerbated” (score of −2). The 6‐point Physician Global Assessment (PGA) score of VA skin lesions was determined quantitatively (0–5) and relatively, and the baseline score (Visit 1) was defined as 4 (Table S5).^7^

**Table 1:** Improvements in vascular anomaly skin lesions (size and color)

Score	Improvements	Criteria
3	Markedly improved	Overall shrinkage, flattening, or disappearance of tumors is observed. A nearly overall large decrease in the intensity of reddishness/purplish or a nearly overall change in reddishness/purplish to the level equal to that of the normal region are obserbed.
2	Improved	Nearly overall shrinkage or flattening of tumors or a nearly overall decrease in the intensity of reddishness/purplish are observed. Or, partial disappearance of tumors or a partial large decrease in the intensity of reddishness/purplish are observed.
1	Slightly improved	Partial shrinkage or flattening of tumors or a partial decrease in the intensity of reddishness/purplish are observed. Or, a nearly overall slight decrease in the intensity of reddishness/purplish is observed.
0	Unchanged	There is no definite change in the size or the reddishness/purplish of tumors.
−1	Slightly exacerbated	Partial enlargement or new formation of tumors or a partial increase in the intensity of reddishness/purplish are observed. Or, a nearly overall slight increase in the intensity of reddishness/purplish is observed.
−2	Exacerbated	A nearly overall enlargement or new formation of tumors or a partial large enlargement of tumors and a partial large increase in the intensity of reddishness/purplish are observed. Or, more severe exacerbation is observed.

Note

Overall, no less than approximately 75% of the extent of the lesion at baseline; nearly overall, approximately 50–75% of the extent of the lesion at baseline; partial, approximately 25–50% of the extent of the lesion at baseline; (the color intensity is) largely decreased, change of three levels or more in red/purple coloration in terms of Pantone Color Sample (Tables S1 and S2); (the color intensity is) decreased/increased, change of two levels or more in red/purple coloration in terms of Pantone Color Sample; (the color intensity is) slightly decreased/increased, change of one level in red/purple coloration in terms of Pantone Color Sample.

We compared the clinical scores evaluated by external evaluators with the change in lesion size and color and performed a correlation coefficient analysis. Primary end‐points included the correlation between the improvement in VA skin lesions (size and color), change in lesion size (volume), and degree of red or purple coloration (Pantone Color Sample) (co‐primary end‐points). Secondary end‐point correlations were as follows: improvements in VA skin lesions (size and color) and change in skin lesion color; improvements in VA skin lesions (size) and change in lesion size; improvements in VA skin lesions (color) and change in the degree of red or purple coloration; improvements in VA skin lesions (sum of size and color scores) and change in lesion size and coloration; 6‐point PGA score of VA skin lesions and change in skin lesion size and color; intraclass correlation coefficients (ICC) (2,1) between external evaluators 1 and 2; and the difference in evaluated scores between the treated and untreated groups.

### 2.3 Statistical analysis

Continuous variables are summarized as median with interquartile range. Categorical variables are summarized as numbers with percentages. Spearman’s correlation coefficients were calculated to compare the objective data of patients with the results measured by two external evaluators. ICC using the two‐way random model (2,1) of each score were calculated to assess inter‐rater reliability. A *p*‐value of less than 0.05 was considered statistically significant. All analyses were performed using the DescTools and irr packages in R version 4.0.3 (The R Foundation for Statistical Computing).

## 3 RESULTS

Between 1 January 2015 and 31 December 2020, 23 cases of VA (seven VT, five LM, three VM, and eight lymphatic–venous malformations) were examined. The characteristics of enrolled patients are shown in Table 2. The median age of patients was 1.0 year (0–24). The lesion locations varied, but the lower extremity was most frequently affected (48%). Sixteen patients were treated with oral sirolimus (≥1) during the observation period. No patients were treated with other therapies.

**Table 2:** Characteristics of patients with vascular anomalies

Characteristic	n = 23
Age (years), median [IQR]	1 [0–8]
Sex, n (%)	
Female	14 (61%)
Male	9 (39%)
Type of vascular anomalies, n (%)	
Vascular tumors	7 (30.4%)
KHE	3 (13%)
TA	4 (17%)
LM	5 (21.7%)
VM	3 (13%)
BRBNS	1 (4.3%)
Combined vascular anomaly **(**LVM)	8 (35%)
Location of the lesion, n (%)	
Upper extremity	3 (13%)
Lower extremity	11 (48%)
Body trunk	6 (26%)
Head region	2 (8.7%)
Gluteal region	1 (4.3%)
Treatment	
Oral sirolimus	16 (70%)
None	7 (30%)

Abbreviations: BRBNS, blue rubber bleb nevus syndrome; IQR, interquartile range; KHE, kaposiform hemangioendothelioma; LM, lymphatic malformation; LVM, lymphatic–venous malformation; TA, tufted angioma; VM, venous malformation.

The measurement outcomes of patients are shown in Table 3. The median skin lesion volume of all patients decreased, and the median degree of red or purple coloration (Pantone Color Sample) also decreased at Visit 2. The value for color tone calculated using image analysis software (Image J) increased, reflecting an improvement in the intensity of red or purple coloration. The median improvement values (VA skin lesion size and color, size, and color) measured by two external evaluators were 1, 0, and 1, respectively. Regarding the co‐primary end‐point, the correlation coefficients between the improvements in VA skin lesions (size and color) and the change in lesion size (volume) and the degree of red or purple coloration (Pantone Color Sample) were −0.695 and −0.869, respectively, and the 95% confidence intervals (CI) were −0.86 to −0.396 (*p* < 0.001) and −0.944 to −0.713 (*p* < 0.001), respectively (Table 4). Additionally, the correlation coefficient between the VA skin lesion improvement score (size and color) and change in color tone score (Image J) was 0.442, and the 95% CI was −0.037 to −0.723 (*p* = 0.035). As for divided scores, the VA skin lesion improvement score (size) correlated with the change in lesion size, and scores (color) were also correlated with a change in the degree of red or purple coloration (Pantone Color Sample) and color (Image J). The change in quantitative 6‐point PGA score and the relative 6‐point PGA score correlated with changes in objective patient data, excluding the change in quantitative 6‐point PGA score and the change in color (Image J). When comparing treated with untreated patients, skin lesion volume and the degree of red or purple coloration in treated patients decreased significantly, whereas the lesion color tone in treated patients improved more markedly compared with untreated patients (Table S6).

**Table 3:** Measurement outcome and patient assessment by external evaluators

Measurement outcome, median [IQR]	Visit 1 (baseline)	Visit 2
Volume of the skin lesion (mm^3^)	200 000 [24 500–600 000]	50 000 [16 800–130 000]
Change of volume (mm^3^)	−12 200 [−456 600 to 0]	
Levels of reddishness or purplish quantitatively (attending doctors, Pantone Color Sample)	4.00 [3.5–5]	2.00 [2–3.5]
Change of the levels of reddishness or purplish quantitatively	−2 [−2.5 to 0]	
Value of color tone (image analysis software, ImageJ)	0.661 [0.485–0.757]	0.718 [0.598–0.779]
Change of value of color tone	0.06 [−0.02 to 0.17]	
Assessment of external evaluators, median [IQR]		
Improvements in skin lesion of vascular anomalies (size and color)		1 [0–2]
Improvements in skin lesion of vascular anomalies (size)		0 [0–0.5]
Improvements in skin lesion of vascular anomalies (color)		1 [0–2]
Improvements in skin lesion of vascular anomalies (sum of size and color scores)		1 [0–2.5]
6‐point PGA	2 [1.5–2.75]	1.50 [1.5–2.25]
Change of 6‐point PGA		0 [−0.25 to 0]
Relative 6‐point PGA score (the baseline score was 4)		3.5 [2.25–4]

Abbreviations: IQR, interquartile range; PGA; Physician Global Assessment.

**Table 4:** Spearman’s correlation coefficient of each statistical analysis

Change of objective patient’s data	Scores measured by the external evaluators					
	Scores of improvements in skin lesion of VA (size and color)	Scores of improvements in skin lesion of VA (size)	Scores of improvements in skin lesion of VA (color)	Sum of scores (size and color)	Change of quantitative 6‐point PGA score	Relative 6‐point PGA score
Change of size (volume)	−0.695 [−0.86 to −0.396] (*p* < 0.001)	−0.262 [−0.609 to 0.168] (*p* < 0.001)	–	−0.587 [−0.805 to −0.231] (*p* = 0.003)	0.423 [0.014–0.711] (*p* = 0.044)	0.617 [0.274–0.82] (*p* = 0.002)
Change of the levels of reddishness or purplish (Pantone Color Sample)	−0.869 [−0.944 to −0.713] (*p* < 0.001)	–	−0.87 [−0.944 to −0.714] (*p* < 0.001)	−0.76 [−0.893 to 0.506] (*p* < 0.001)	0.638 [0.307–0.832] (*p* = 0.001)	0.818 [0.612–0.92] (*p* < 0.001)
Change of color value (image analysis software, ImageJ)	0.442 [0.037–0.723] (*p* < 0.001)	–	0.44 [0.034–0.722] (*p* = 0.035)	0.517 [0.133–0.766] (*p* = 0.012)	−0.293 [−0.629 to 0.136] (*p* = 0.175)	−0.534 [−0.776 to −0.157] (*p* = 0.009)

Note

Statistical data are shown as Spearman’s correlation coefficients, [IQR], and *p*‐value.

Abbreviations: IQR, interquartile range; PGA, Physician Global Assessment; VA, vascular anomaly.

Two independent external evaluators determined scores from clinical photographs only at Visits 1 and 2. ICC (2,1) of VA skin lesion improvement score (size and color), VA size, and VA color between external evaluators 1 and 2 were 0.776, 0.799, and 0.828, respectively, and 95% CI were 0.544–0.898, 0.572–0.911, and 0.638–0.923, respectively (*p* < 0.001 for all).

## 4 DISCUSSION

We conducted a validation study to establish methods to evaluate treatment outcomes in patients with VA skin lesions. We used three measurement scores (improvements in VA skin lesions) and 6‐point PGA scores of VA skin lesions and analyzed the correlation between these scores and actual objective data. The data suggest that measurement scores evaluated from patients’ photographs were effective at expressing changes in lesion size and color. Additionally, ICC values between external evaluators 1 and 2 showed high reliability. To our knowledge, this is the first study to assess the responsiveness of VA skin lesion measurement scores.

In the current literature, no uniform assessment methods evaluate the effectiveness of treatment for VA skin lesions. Therefore, treatment outcomes in different clinical trials cannot be compared. mTOR plays a key role in the pathogenesis of various VA, and the mTOR inhibitor sirolimus has been identified as a promising treatment for VA.^3^ In a recent systematic review of sirolimus treatment for VA, Freixo et al.^5^ described that the definition and assessment of outcomes were heterogeneous between studies, and were thus difficult to compare systematically. In the first large‐sample study, Adams et al.^3^ showed a high response rate to sirolimus in patients with LA. They used three distinct assessments, including radiological examination, functional impairment score, and QOL score. Triana et al.^4^ also reported 41 patients with VA who displayed volume reduction with sirolimus treatment by radiological imaging. In patients with LM, our clinical trial attempted to assess not only lesion volume using magnetic resonance imaging, but also the severity of VA to assess the degree of impairment of affected organs.^8^ These clinical scores are very useful to assess organ function in patients with VA, but would be unsuitable to evaluate VA skin lesions. At present, no validated measurement scores for VA skin lesions are available.

Clinical assessments using photographs of skin lesions have been widely used in dermatology. In clinical trials of IH treated with propranolol, digital photographs of skin lesions were taken before and after treatment.^9^ The photographs included a color chart to calibrate color and size, and were assessed by two independent and trained readers using a photograph‐based centralized assessment. The assessment criteria for treatment depended on the degree of target lesion resolution after propranolol treatment. Furthermore, the latest clinical trials of voluminous and complicated superficial slow‐flow vascular malformations with sirolimus (PERFORMUS) and topical sirolimus 0.1% for treating cutaneous microcystic LM in children and adults (TOPICAL) are planning to assess digital photographs at baseline and after treatment.^10^, ^11^ In these studies, photographs will be qualitatively assessed by two blinded independent trained readers without informative data. However, the details of these evaluation methods and criteria of ongoing these trials have not yet been officially announced.

In this study, we used a novel measurement score to evaluate the efficacy of drugs in patients with VA, which was referenced to the outcome score of improvements in facial angiofibroma in patients with TSC. This score is useful and easy to assess.^6^ The outcome measurement score of angiofibroma was also assessed by independent evaluators using photographs. We created three distinct scores, including a combined score for size and color, size alone, and color alone. Our results reveal that all measurement scores evaluated by external evaluators correlated with actual objective data in all patients, including treated patients. This suggests that our VA scoring system could be useful for evaluating treatments for skin lesions.

Of note, the 6‐point PGA scoring system is a frequently used primary end‐point in dermatology clinical trials.^7^ It is known that evaluating this score using clinical photographs is useful.^12^ In an ongoing phase 2, randomized, double‐blind, controlled clinical trial of topical sirolimus for microcystic LM, the 6‐point PGA score will be used to evaluate cutaneous microcystic LM as a primary outcome.^11^ Although the score is not specified in skin VA, our results show excellent correlation between scores evaluated by two external evaluators and patients’ objective data. This suggests that 6‐point PGA may also be a useful measurement scoring system as an alternative end‐point.

Our study had several limitations. This was a retrospective study, and the number of patients was small. The study included patients with a variety of VA and heterogeneous diseases. Thus, we should consider differences between these diseases in future research.

In conclusion, we validated measurement scores to evaluate treatment for VA skin lesions. External assessments using our modified scores were significantly correlated with actual objective changes in patient data. Our results provide novel measurement scores to evaluate the efficacy of treatments for VA skin lesions.

## CONFLICT OF INTEREST

M.O. and H.O. received research funding from Nobelpharma. Sirolimus was supplied by Nobelpharma (Nobelpharma Co., Ltd., Tokyo, Japan). The other authors declare no competing interests.

## Supporting information

Supplementary Material
