# Supplementary material for: Validation of measurement scores for evaluating vascular anomaly skin lesions
Source: J Dermatol. 2021 Mar 30;48(7):993–8. doi: 10.1111/1346-8138.15839 (PMC8360112; doi:10.1111/1346-8138.15839)
Supplement: Supplementary file 1 — Supplementary Material [file JDE-48-993-s001.docx]

**Supplemental Tables**

a. Status of red coloration in terms of Pantone^®︎^ Color Sample.

| Level | Status of Reddishness | Pantone^®︎^ Color Sample |
| --- | --- | --- |
| 1 | As dark as or paler than Pantone^®︎^ 489C | 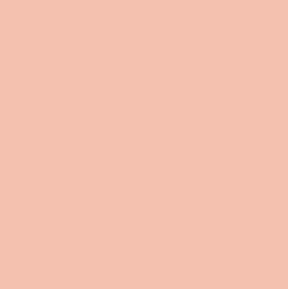 |
| 2 | As dark as or paler than Pantone^®︎^ 486C | 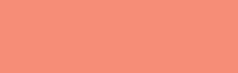 |
| 3 | As dark as or paler than Pantone^®︎^ 7416C | 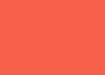 |
| 4 | As dark as or paler than Pantone^®︎^ 485C | 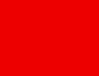 |
| 5 | As dark as or paler than Pantone^®︎^ 704C | 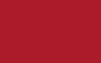 |
| 6 | Darker than Pantone^®︎^ 704C | - |

The colors shown in the Pantone^®︎^ Color Sample column in the above table are not an accurate representation of the color tones indicated by the numbers. When assessing red coloration, always use a sample for assessing the degree of red coloration.

b. Status of purple coloration in terms of Pantone^®︎^ Color Sample.

| Level | Status of Purplish | Pantone^®︎^ Color Sample |
| --- | --- | --- |
| 1 | As dark as or paler than Pantone^®︎^ 263C | 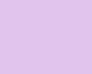 |
| 2 | As dark as or paler than Pantone^®︎^ 264C | 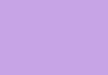 |
| 3 | As dark as or paler than Pantone^®︎^ 265C | 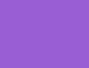 |
| 4 | As dark as or paler than Pantone^®︎^ 266C | 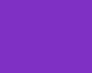 |
| 5 | As dark as or paler than Pantone^®︎^ 2607C | 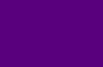 |
| 6 | Darker than Pantone^®︎^ 2607C | - |

The colors shown in the Pantone^®︎^ Color Sample column in the above table are not an accurate representation of the color tones indicated by the numbers. In the assessment of purple coloration, always use a sample to assess the degree of purple coloration.

c. Improvements in vascular anomaly skin lesions (size).

| Score | Improvements | Criteria |
| --- | --- | --- |
| 3 | Markedly Improved | Overall shrinkage, flattening, or disappearance of tumors is observed. |
| 2 | Improved | Nearly overall shrinkage or flattening of tumors is observed. Or, partial disappearance of tumors is observed. |
| 1 | Slightly improved | Partial shrinkage or flattening of tumors is observed. |
| 0 | Unchanged | There is no definite change in the size of tumors. |
| -1 | Slightly exacerbated | Partial enlargement or new formation of tumors is observed. |
| -2 | Exacerbated | A nearly overall enlargement or new formation of tumors or a partial large enlargement of tumors is observed. Or, more severe exacerbation is observed. |

Overall, no less than approximately 75% of the extent of the lesion at baseline; nearly overall, approximately 50%–75% of the extent of the lesion at baseline; partial, approximately 25%–50% of the extent of the lesion at baseline.

d. Improvements in vascular anomaly skin lesions (color).

| Score | Improvements | Criteria |
| --- | --- | --- |
| 3 | Markedly Improved | A nearly overall large decrease in the intensity of reddishness/purplish is observed. Or, a nearly overall change in reddishness/purplish to the level equal to that of the normal region is observed. |
| 2 | Improved | A nearly overall decrease in the intensity of reddishness/purplish is observed. Or, a partial large decrease in the intensity of reddishness/purplish is observed. |
| 1 | Slightly improved | A nearly overall slight decrease in the intensity of reddishness/purplish is observed. Or, a partial decrease in the intensity of reddishness/purplish is observed. |
| 0 | Unchanged | There is no definite change in the reddishness/purplish of tumors. |
| -1 | Slightly exacerbated | A nearly overall slight increase in the intensity of reddishness/purplish is observed. Or, a partial increase in the intensity of reddishness/purplish is observed. |
| -2 | Exacerbated | A nearly overall increase in the intensity of reddishness/purplish or a partial large increase in the intensity of reddishness/purplish is observed. Or, more severe exacerbation is observed. |

Overall, no less than approximately 75% of the extent of the lesion at baseline; nearly overall, approximately 50%–75% of the extent of the lesion at baseline; partial, approximately 25%–50% of the extent of the lesion at baseline; (the color intensity is) largely decreased, change of three levels or more in red/purple coloration in terms of Pantone^®︎^ Color Sample (Supplemental Tables a and b); (the color intensity is) decreased/increased: change of two levels or more in red/purple coloration in terms of Pantone^®︎^ Color Sample; (the color intensity is) slightly decreased/increased, change of one level in red/purple coloration in terms of Pantone^®︎^ Color Sample.

e. Improvements in vascular anomaly skin lesions (sum of size and color scores).

The total vascular anomaly skin lesion improvement score (size from 3 to −2 and color from 3 to −2). There are 11 scores (from 6 to −2).

f. Six-point Physician Global Assessment (PGA) score of vascular anomaly skin lesions.

| Score | Improvements | Criteria |
| --- | --- | --- |
| 0 | Clear | No sign of vascular anomalies |
| 1 | Almost clear | Only minimal or almost clear of vascular anomalies |
| 2 | Mild | Mild sign of vascular anomalies |
| 3 | Moderate | Moderate sign of vascular anomalies |
| 4 | Severe | Severe sign of vascular anomalies |
| 5 | Very severe | Very severe sign of vascular anomalies |

g. Characteristics of treated and untreated patients with VAs.

| Characteristic | Treated (N = 16) | | Untreated (N = 7) | | |
| --- | --- | --- | --- | --- | --- |
| Type of vascular anomalies, No. (%) |  | |  | | |
| Vascular tumors | 5 (31.3%) | | 2 (28.6%) | | |
| KHE | 3 (18.8%) | | 0 | | |
| TA | 2 (12.5%) | | 2 (28.6%) | | |
| LM | 5 (21.7%) | | 0 | | |
| VM | 1 (6.3%) | | 2 (28.6%) | | |
| BRBNS | 1 (6.3%) | | 0 | | |
| Combined vascular anomaly (LVM) | 5 (31.3%) | | 3 (42.9%) | | |
|  | | |  | | |
| Location of the lesion, No. (%) | | |  | | |
| Upper extremity | 2 (12.5%) | | 1 (14.3%) | | |
| Lower extremity | 7 (43.8%) | | 4 (57.1%) | | |
| Body trunk | 4 (25%) | | 2 (28.6%) | | |
| Head region | 2 (8.7%) | | 0 | | |
| Gluteal region | 1 (4.3%) | | 0 | | |
|  |  | |  | | |
| The measurement outcome, median [IQR] | Visit 1 (baseline) | Visit 2 | Visit 1  (baseline) | | Visit 2 |
| Volume of the skin lesion (mm^3^) | 325,000 [30,125, 1,000,000] | 43,000 [2,450, 106,850] | 105,000 [24,500, 300,000] | | 52,500 [24,500, 360,000] |
| **The change of volume (mm^3^)** | **-73,500 [-599,400, -1,006.25]** | | **0 [0, 0]** | | |
| The levels of reddishness or purplish quantitatively (Pantone^®︎^ Color Sample) | 5 [3.75, 5] | 2 [2, 2.25] | 4 [3.5, 4] | | 4 [3, 4] |
| **The change of the levels of reddishness or purplish quantitatively** | **-2 [-3, -1]** | | **0 [0, 0]** | | |
| The value of color tone (image analysis software, ImageJ) | 0.662 [0.485, 0.756] | 0.731 [0.664, 0.894] | 0.619 [0.589, 0.774] | | 0.674 [0.579, 0.752] |
| **The change of value of color tone** | **0.12 [-0.01, 0.19]** | | **0.02 [-0.04, 0.05]** | | |
|  |  | |  | | |
|  | Visit 1 | Visit 2 | Visit 1 | Visit 2 | |
| 6-point PGA | 2 [1.5, 2.62] | 1.75 [1.5, 2.12] | 1.5 [1.5, 2.5] | 1.5 [1.5, 2.5] | |
| **The change of 6-point PGA** | **0 [-0.5, 0]** | | **0 [0, 0]** | | |
| The　relative　6-point PGA score (the baseline score was 4) | 3 [2, 3.62] | | 4 [3.75, 4] | | |

Abbreviations: KHE, kaposiform hemangioendothelioma; TA, tufted angioma; LM, lymphatic malformation; VM, venous malformation; BRBNS, blue rubber bleb nevus syndrome; LVM, lymphatic–venous malformation; PGA, Physician Global Assessment; IQR, interquartile range.
